# Application of Artificial Intelligence in Early Diagnosis of Spontaneous Preterm Labor and Birth

**DOI:** 10.3390/diagnostics10090733

**Published:** 2020-09-22

**Authors:** Kwang-Sig Lee, Ki Hoon Ahn

**Affiliations:** 1 AI Center, Korea University Anam Hospital, Seoul 02841, Korea; ecophy@hanmail.net; 2 Department of Obstetrics & Gynecology, Korea University Anam Hospital, Seoul 02841, Korea

**Keywords:** preterm birth, early diagnosis, artificial intelligence

## Abstract

This study reviews the current status and future prospective of knowledge on the use of artificial intelligence for the prediction of spontaneous preterm labor and birth (“preterm birth” hereafter). The summary of review suggests that different machine learning approaches would be optimal for different types of data regarding the prediction of preterm birth: the artificial neural network, logistic regression and/or the random forest for numeric data; the support vector machine for electrohysterogram data; the recurrent neural network for text data; and the convolutional neural network for image data. The ranges of performance measures were 0.79–0.94 for accuracy, 0.22–0.97 for sensitivity, 0.86–1.00 for specificity, and 0.54–0.83 for the area under the receiver operating characteristic curve. The following maternal variables were reported to be major determinants of preterm birth: delivery and pregestational body mass index, age, parity, predelivery systolic and diastolic blood pressure, twins, below high school graduation, infant sex, prior preterm birth, progesterone medication history, upper gastrointestinal tract symptom, gastroesophageal reflux disease, Helicobacter pylori, urban region, calcium channel blocker medication history, gestational diabetes mellitus, prior cone biopsy, cervical length, myomas and adenomyosis, insurance, marriage, religion, systemic lupus erythematosus, hydroxychloroquine sulfate, and increased cerebrospinal fluid and reduced cortical folding due to impaired brain growth.

## 1. Introduction

### 1.1. Preterm Birth

Preterm birth is a major cause of disease burden for newborns and children in the world [1,2,3,4]. Preterm birth affected one of every 10 newborns in the US during 2003–2012, i.e., 5,042,982 (12.2%) of 41,206,315 births [1]. Every year 15 million births are estimated to be preterm on a global level and preterm birth is the leading cause of neonatal and childhood mortality in the world, responsible for 1 million deaths among those aged less than five years [2,3]. Indeed, it is reported that 75% of this mortality can be prevented with cost-effective interventions [4]. Preterm birth is defined as birth occurring at less than 37 weeks of gestational age and it can be divided into three types, e.g., spontaneous labor with intact membranes (or spontaneous preterm labor and birth) (40–45%), preterm premature rupture of the membranes (25–30%), and labor induction or caesarean delivery for maternal or fetal indications (30–35%) [5]. This study focuses on spontaneous preterm labor and birth (“preterm birth” hereafter), which takes the largest proportion. The factors of preterm birth are unknown generally but existing literature considers the following maternal variables to be its major determinants: socioeconomic status such as education, income, workload; health conditions including body mass index, hypertensive disorder, diabetes mellitus; and other obstetric variables including cesarean section, infection, in vitro fertilization or intracytoplasmic sperm injection pregnancy, parity, placenta abruption, placenta previa, prior abortion, prior preterm birth, short cervix, and vaginal bleeding [5,6,7,8,9,10,11,12,13]. 

### 1.2. Artificial Intelligence

**Figure 1:**
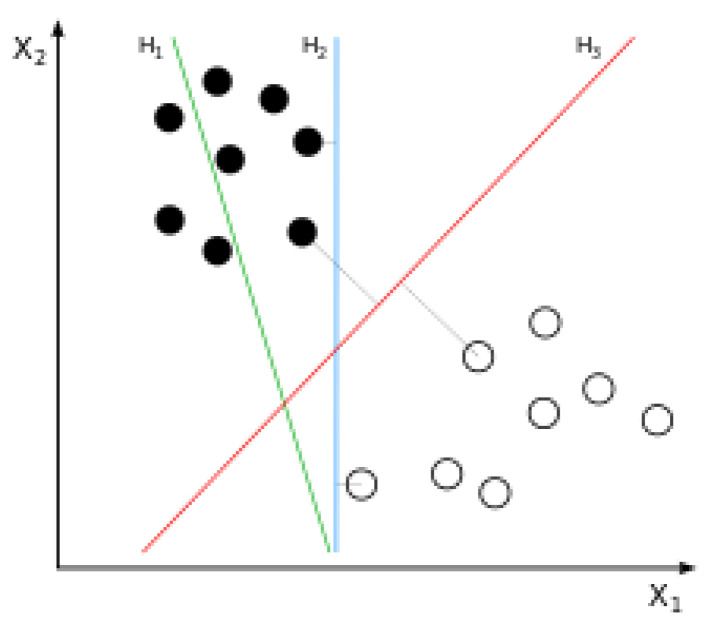
Principle of the support vector machine. A line (or hyperplane) *H*_1_ does not separate black and white circles while hyperplanes *H*_2_ and *H*_3_ separate the two groups. Here, the distance between the two groups is maximal for *H*_3_ and the support vector machine creates such a hyperplane. Image provided by Wikipedia.

Recently, the words “artificial intelligence”, “machine learning”, and “deep learning” have gained strong interest around the world. For example, the Google trends for these keywords registered rapid growths from 10 to 100 from 2013 to 2018. The Merriam-Webster dictionary defines artificial intelligence as “the capability of a machine to imitate intelligent human behavior”. Machine learning (or data mining) is a branch of artificial intelligence to “extract knowledge from large amounts of data” [14]. Six popular machine learning methods are the decision tree, the naïve Bayesian classifier, the random forest, the support vector machine, the artificial neural network, and the deep neural network (deep learning). A decision tree consists of (1) internal nodes (each meaning a test on an independent variable), (2) branches (each denoting an outcome of the test) and (3) terminal nodes (each representing the dependent variable). A naïve Bayesian classifier is a classifier from Bayes’ theorem. Bayes’ theorem states that the probability of the dependent variable given certain values of independent variables can be derived from the probabilities of the independent variables given a certain value of the dependent variable. The naïve Bayesian classifier makes a prediction based on this theorem. A random forest consists of many decision trees and takes a majority vote on the dependent variable (“bootstrap aggregation”). Let us consider a random forest with 1000 decision trees. In this example, 1000 training sets are sampled with replacements, 1000 decision trees are trained with the 1000 training sets, the 1000 decision trees make 1000 predictions and the random forest takes a majority vote on the dependent variable. A support vector machine creates a line or space (“hyperplane”), which separates data with the maximal distance between different groups [14]. Let us consider Figure 1 as an example. Here, a line (or hyperplane) *H_1_* does not separate black and white circles while hyperplanes *H_2_* and *H_3_* separate the two groups. Here, the distance between the two groups is maximal for *H_3_* and the support vector machine creates such a hyperplane.

**Figure 2:**
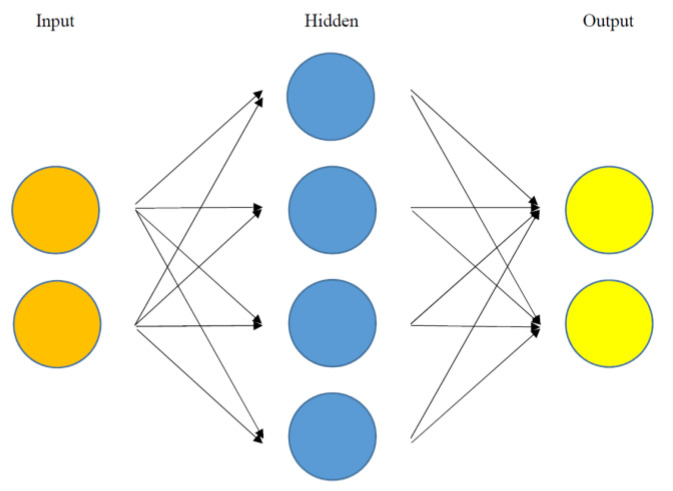
Structure of the artificial neural network. An artificial neural network is a network of input/output units (so called “neurons”) connected through weights. The artificial neural network usually consists of one input layer, one, two or three hidden layers, and one output layer. Neurons in a previous layer combine with “weights” in the next layer (here, the weights are numerical values showing how much effect neurons in a previous layer have on neurons in the next layer).

An artificial neural network is a network of input/output units (so called “neurons”) connected through weights (Figure 2). The artificial neural network usually consists of one input layer, one, two or three hidden layers, and one output layer. Neurons in a previous layer combine with “weights” in the next layer (Here, the weights are numerical values showing how much effect neurons in a previous layer have on neurons in the next layer). This operation is done in the order of weights in a layer next to the input layer, its following layer, and so on. This process is called the feedforward algorithm. Then, these weights are adjusted based on how much contribution they made to the “loss” of the artificial neural network (Here, the loss is a gap between actual and predicted final outputs of the artificial neural network). This operation is done in the order of weights in the output layer, its previous layer, and so on. This process is called the backpropagation algorithm. These algorithms are repeated until a certain standard is achieved for the accurate prediction of the dependent variable [14]. This unique process distinguishes the final results of the artificial neural network from other machine learning methods. Finally, deep learning is a sub-group of the artificial neural network whose number of hidden layers is larger than five, e.g., ten. A vast expansion of computing power has been a major contributor for the unprecedented performance and popularity of the artificial neural network and its deep learning counterparts on a worldwide level over the past decades.

### 1.3. Aims of Study

Conventional studies covered only a limited range of factors for preterm birth, while centering on logistic regression with an unrealistic assumption of *ceteris paribus*, i.e., “all the other variables staying constant”. For this reason, emerging literature started to employ artificial intelligence for various tasks and sectors, for example, classification, prediction and pattern recognition for business, finance, and medicine [15,16,17,18,19,20,21]: They are free from unrealistic assumptions of “all the other variables staying constant” and they can analyze which variables are more important for the classification, prediction or pattern recognition of the dependent variable. This article reviews these studies and addresses future perspectives on this issue. The aim of this study is to review the current status and future prospective of knowledge on the use of artificial intelligence for the prediction of preterm birth. 

### 1.4. Methods of Study

Seven articles were selected for review out of 107 articles in Google and PubMed with the search terms “artificial intelligence” and “spontaneous preterm labor and birth”. The following eligibility criteria were adopted: (1) the intervention(s) of a new deep learning method or several machine learning approaches addressed above (the decision tree, the naïve Bayesian classifier, the random forest, the support vector machine, the artificial neural network, and/or the deep neural network); (2) the outcome(s) of accuracy and/or the area under the receiver operating characteristic curve for the prediction of spontaneous preterm labor and birth; (3) the publication year of 2000 or later; and (4) the publication language of English. The following summary measures were employed: artificial intelligence method, sample size, data type, performance measures, and important predictors (independent variables). Here, accuracy denotes the proportion of correct predictions over all observations, whereas the area under the receiver operating characteristic curve represents the plot of the true positive rate (sensitivity) against the false positive rate (1-specificity) at various threshold settings. 

## 2. Application of Machine Learning in Early Diagnosis of Spontaneous Preterm Labor and Birth

### 2.1. Duke University Medical Center Study

The artificial neural network and the random forest have been known for their performance that is comparable or superior to those of traditional methods such as logistic regression regarding the prediction of preterm birth [17,18,19,20,21]. For example, a recent study used data with 19,970 participants at the Duke University Medical Center from 1 January 1988 to 1 June 1997 [17,18,19]. The dependent variable (or the class) was preterm birth and the independent variables (or the attributes) were 1622 demographic, obstetric variables including age, race (Asian, black, white, Hispanic, American Indian, unknown), region (Durham, Orange, unknown), religion (Catholic, Jewish, Protestant, other), education (graduate school, college, technical school, high school, grade school, unknown), insurance (private, public, other), and marital status (married, divorced, separated, single, widowed, unknown). The artificial neural network showed a better performance than logistic regression and the decision tree: the areas under the receiver operating characteristic curves were 0.66, 0.65, and 0.68 for logistic regression, the decision tree, and the artificial neural network, respectively.

### 2.2. Korea University Anam Hospital Study

Another recent study made a rare attempt to compare popular machine learning methods for the analysis of preterm birth [20]. Data for this study consisted of 596 obstetric patients in Anam Hospital (Seoul, Korea) during 27 March 2014–21 August 2018. The dependent variable was preterm birth and the independent variables were: demographic status such as age, health conditions including body mass index, drinker (no, yes), smoker (no, yes), diabetes mellitus (no, yes), hypertensive disorder (no, yes), and other obstetric variables including cervical length measured between 18 and 24 weeks of gestation (cm), in vitro fertilization (no, yes), myomas and adenomyosis (no, yes), parity, pelvic inflammatory disease history (no, yes), prior cone biopsy (no, yes), prior placenta previa (no, yes), and prior preterm birth (no, yes). The artificial neural network, logistic regression, and the random forest were trained based on 298 participants (training set) and validated based on the other 298 participants (validation set). Variable importance (an accuracy gap between a complete model and a model excluding a certain variable) was employed to identify major factors of preterm birth. In terms of accuracy, the artificial neural network (0.9115) was comparable to logistic regression and the random forest (0.9180 and 0.8918, respectively). The variable importance results of the artificial neural network and the random forest requested due attention to body mass index, hypertension, diabetes mellitus, prior cone biopsy, parity, cervical length, age, prior preterm birth, and myomas and adenomyosis. However, the artificial neural network outcomes placed more emphasis on hypertension, diabetes mellitus, and prior cone biopsy whereas their random forest counterparts put more focus on cervical length, age and prior preterm birth. The artificial neural network outcomes were consistent with several previous studies [22,23,24,25,26,27,28,29,30,31,32,33], suggesting the shift of focus from direct determinants of preterm birth to their indirect counterparts.

A more recent study used similar approaches for analyzing preterm birth, gastroesophageal reflux disease, and periodontitis [21]. Previous studies made independent suggestions on a positive association between gastroesophageal reflux disease and periodontitis and on a positive linkage between periodontitis and preterm birth [34,35,36,37,38,39,40]. In a similar vein, one would suggest a positive relationship between gastroesophageal reflux disease and preterm birth. However, no literature was available on this topic. Data for this study were made of 731 obstetric patients at Anam Hospital (Seoul, Korea) from 5 January 1995 until 28 August 2018. The dependent variable was preterm birth and the following independent variables were included in this study: (1) demographic/socioeconomic status such as age, below high school graduation (no, yes), public insurance/Medicaid only (no, yes), urban area (no, yes); (2) health conditions including pregestational and delivery body mass index, drinking (no, yes), smoking (no, yes), type I, type II and gestational diabetes mellitus (no vs. yes for each type), predelivery systolic and diastolic blood pressure (mmHg), chronic and gestational hypertension (no vs. yes for each type), periodontitis (no, yes), upper gastrointestinal tract symptom (no, yes), gastroesophageal reflux disease (no, yes), Helicobacter pylori (no, yes); and (3) other obstetric variables including adenomyosis (no, yes), in vitro fertilization (no, yes), infant sex (male, female), myoma uteri (no, yes), parity, pelvic inflammatory disease history (no, yes), preeclampsia (no, yes), prior cone (no, yes), prior preterm birth (no, yes), prior previa (no, yes), twin (no, yes), medication history (no vs. yes for each of progesterone, calcium channel blocker, nitrate, tricyclic antidepressant, benzodiazepine, and sleeping pills).

The random forest (0.8681) registered similar accuracy with logistic regression (0.8736). The variable importance results of the random forest highlighted the significance of delivery and pregestational body mass index, age, parity, predelivery systolic and diastolic blood pressure, twins, below high school graduation, infant sex, prior preterm birth, progesterone medication history, upper gastrointestinal tract symptom, gastroesophageal reflux disease, Helicobacter pylori, urban region, calcium channel blocker medication history, and gestational diabetes mellitus. As a matter of fact, pregnant women often do not pay much attention to gastroesophageal reflux disease-related symptoms such as nausea and vomiting, given that these common symptoms do not look severe. A clinical implication of the statistical results above, however, states that more active care is essential for these common symptoms to prevent more severe outcomes including preterm birth. Another clinical implication from this study states that preventive measures for diabetes mellitus, hypertensive disorder, and gastroesophageal reflux disease would be useful for the prevention of preterm birth, together with effective management of body mass index and progesterone and calcium channel blocker medications.

### 2.3. U.S. Center for Disease Control Study

The most recent endeavor on this topic involved the use of machine learning for predicting early stillbirth, late stillbirth, and preterm birth [41]. The training set for this study consisted of 15,976,537 pregnancies from 2013 to 2016 enrolled in the Center for Disease Control in the United States. The validation set was made of 364,124 pregnancies from 2013 to 2016 enrolled in the New York City Department of Health and Mental Hygiene. The dependent variables were early stillbirth, late stillbirth, and preterm birth. Here, early (or late) stillbirth was defined as the birth of a baby with no signs of life after 22 (or 28) weeks of pregnancy. The independent variables on demographic characteristics were age (years), race (white, black, Alaskan/Native American, Asian), marital status (no, yes), education (8th grade or less, 9th–12th with no diploma, high school diploma, college credit with no degree, associate degree, bachelor degree, master degree, doctoral/professional degree), previous terminations, Special Nutritional Program for Women, Infants and Children (no, yes), pre-pregnancy smoking (0, 1–5, 6–10, 11–20, 21–40, 41–), body mass index, and weight (pounds). The following obstetric independent variables were also included: parity (nulliparous, parous), pre-pregnancy diabetes (no, yes), gestational diabetes (no, yes), pre-pregnancy hypertension (no, yes), gestational hypertension (no, yes), hypertension eclampsia (no, yes), previous preterm birth (no, yes), infertility treatment (no, yes), infertility medication (no, yes), Assisted Reproductive Technology, previous cesarean section (no, yes), gonorrhea (no, yes), syphilis (no, yes), chlamydia (no, yes), hepatitis B (no, yes), and hepatitis C (no, yes).

Four machine learning approaches (logistic regression, the gradient boosting decision tree, and artificial neural networks with the activation functions of rectified and scaled exponential linear units) were applied for predicting early stillbirth, late still birth, and preterm birth in this study. The gradient boosting decision tree reduces the bias of a decision tree ensemble based on residual fitting in a sequential manner. The artificial neural networks with the activation functions of rectified and scaled exponential linear units were designed to solve the problem of gradient vanishing (the gradient of the loss with respect to the weight becomes 0 quickly). The respective sensitivity measures of the four methods were 0.22, 0.24, 0.24, and 0.24 for preterm birth. The respective areas under the receiver operating characteristic curves of the four methods were 0.62, 0.63, 0.64, and 0.64 for preterm birth (the artificial neural networks showed the best performance). Based on the results of logistic regression for predicting preterm birth in this study, the coefficients of all independent variables except hepatitis B were statistically significant at 1%.

### 2.4. Ljubljana University Medical Center Study

Another notable attempt was made recently on the effective application of machine learning on the prediction of preterm birth using uterine electrohysterogram data [42]. Data for this study were made of 300 obstetric patients (38 preterm) at the University Medical Centre Ljubljana (Slovenia) from 1997 to 2005. The training and validation sets for this study consisted of 240 and 60 pregnancies, respectively. The dependent variable was preterm birth and the independent variables were root mean squares, peak frequency, median frequency, and sample entropy extracted from the electrohysterogram data. Nine machine learning methods were applied and compared for the prediction of preterm birth and the support vector machine showed the best performance in terms of specificity (1.0000) and the area under the receiver operating characteristic curve (0.61). A more recent study reached a similar conclusion on the best performance of the support vector machine regarding the prediction of preterm birth using electrohysterogram data [43]. The dependent variable was preterm birth and the independent variables were twelve intrinsic mode functions from the Huang–Hilbert transformation. The support vector machine achieved the accuracy, sensitivity, specificity, and area under the receiver operating characteristic curve of 0.92, 0.93, 0.92, and 0.96, respectively. 

## 3. Application of Deep Learning in Early Diagnosis of Spontaneous Preterm Labor and Birth

As defined above, deep learning is a sub-group of the artificial neural network whose number of hidden layers is larger than five, e.g., ten. Different types of deep learning have been designed for different types of data, e.g., the convolutional neural network for image data and the recurrent neural network for sequence data such as electronic health records. In the convolutional neural network, a feature detector (“kernel”) moves across input data and the dot product of its elements and their input data counterparts is calculated. This operation of “convolution” identifies certain characteristics of the input data, for example, the shape of a tumor compared to that of a normal cell [44]. In the recurrent neural network, the current output information depends, in a repetitive (or “recurrent”) pattern, on the current input information and the previous hidden state (which is the memory of the network on what happened in all previous periods) [45]. Acquiring big data that is also high quality is essential for these cutting-edge approaches to be effective and, for this reason, there is little or no such endeavor in case big data is not available (as for preterm birth). However, this situation might change and more effort needs to be made in this direction, given an increasing amount of publication on this topic in very recent years [46,47].

For example, a recent study used a recurrent neural network ensemble to predict extreme preterm birth (birth before the 28th week of gestational age) [46]. Data for this study came from electronic health records on 25,689 deliveries at the Vanderbilt University Medical Center. The performance of the recurrent neural network ensemble exceeded those of logistical regression and the support vector machine: 0.965 vs. 0.819 and 0.660 in sensitivity, 0.827 vs. 0.749 and 0.728 in the area under the receiver operating characteristic curve. Based on the results of logistic regression in this study, extreme preterm birth had positive associations with twin pregnancies, short cervical length, hypertensive disorder, systemic lupus erythematosus, and hydroxychloroquine sulfate. In a similar vein, another recent study used a 3-dimensional convolutional neural network with layer-wise relevance propagation to predict preterm birth [47]. Here, layer-wise relevance propagation is a backpropagation algorithm characterized by “the assignment of a relevance score to each input voxel”. The data source of this study was 157 magnetic resonance imaging scans of infants born between 23–42 weeks of gestational age. The performance measures of the convolutional neural network looked very impressive: 94% accuracy, 100% true positive rate, 86% true negative rate. These studies would be good starting points and more research is to be done for the effective application of deep learning for early diagnosis of preterm birth.

## 4. Summary of Study

The summary of review is presented in Table 1. The table includes five summary measures such as artificial intelligence method, sample size, data type, performance measures, and important predictors (independent variables). The summary of review suggests that different machine learning approaches would be optimal for different types of data regarding the prediction of preterm birth: the artificial neural network, logistic regression and/or the random forest for numeric data, the support vector machine for electrohysterogram data, the recurrent neural network for text data, and the convolutional neural network for image data. Here, the ranges of performance measures were 0.79–0.94 for accuracy, 0.22–0.97 for sensitivity, 0.86–1.00 for specificity, and 0.54–0.83 for the area under the receiver operating characteristic curve. The summary of review also indicates that the following maternal variables can be considered to be major determinants of preterm birth: delivery and pregestational body mass index, age, parity, predelivery systolic and diastolic blood pressure, twins, below high school graduation, infant sex, prior preterm birth, progesterone medication history, upper gastrointestinal tract symptom, gastroesophageal reflux disease, Helicobacter pylori, urban region, calcium channel blocker medication history, gestational diabetes mellitus, prior cone biopsy, cervical length, myomas and adenomyosis, insurance, marriage, religion, systemic lupus erythematosus, hydroxychloroquine sulfate, and increased cerebrospinal fluid and reduced cortical folding due to impaired brain growth. However, artificial intelligence is a data-driven approach and more research is needed for more general conclusions. 

## 5. Current Limitations and Future Perspectives

Existing literature on the early diagnosis of preterm birth based on artificial intelligence suffers from several limitations and more improvement is needed in these directions. Firstly, these studies used a cross-sectional design because of limited data availability. Expanding data with a longitudinal design is expected to improve the performance of artificial intelligence much more. Secondly, these studies did not consider possible mediating effects among variables. Thirdly, these studies used data with small sample sizes in single centers. Expanding these studies to big data (such as national health insurance claims data) will be a good topic for future research. Fourthly, the areas under the receiver operating characteristic curves of machine learning methods in these studies (0.54–0.83) might not be optimal as diagnostic tests yet. Fifthly, binary categories (no, yes) are common for preterm birth but their categories can be refined further, for example, previable (less than 24 weeks of gestation), very early (24–27 weeks), early (28–31 weeks), and late (32–37 weeks). Analyzing various factors of preterm birth based on more refined categories will be an interesting issue for future research. Sixthly, little investigation has been carried out and more study is needed on possible pathways between gastroesophageal reflux disease and preterm birth [21]. Seventhly, combining different types of deep learning models for different types of preterm birth data would break new ground and bring more profound clinical insights. Finally, there has been no basic or translational research based on artificial intelligence regarding any type of preterm birth, spontaneous or medically indicated.

This article reviewed the existing literature and addressed the future perspectives regarding the application of artificial intelligence in early diagnosis of spontaneous preterm labor and birth. This review indicates that artificial intelligence provides a potential for a non-invasive tool and decision support system for early diagnosis of spontaneous preterm labor and birth.

## Figures and Tables

**Table 1:** Summary of review.

Publication	Method	Sample Size	Data Type	Performance	Important Predictors
[18]	ANN * DT LR	19,970	Numeric	AUC 0.65–0.68	Age, race, region, religion, education, insurance, marriage
[20]	ANN * DT LR * NB RF * SVM *	596	Numeric	Accuracy 0.89–0.92 AUC 0.62–0.64	Body mass index, hypertension, diabetes mellitus, prior cone biopsy, parity, cervical length, age, prior preterm birth, myomas & adenomyosis **
[21]	ANN DT LR * NB RF * SVM	731	Numeric	Accuracy 0.79–0.87 AUC 0.54–0.76	Delivery and pregestational body mass index, age, parity, predelivery systolic and diastolic blood pressure, twin, below high school graduation, infant sex, prior preterm birth, progesterone medication history, upper gastrointestinal tract symptom, gastroesophageal reflux disease, Helicobacter pylori, urban region, calcium-channel-blocker medication history, gestational diabetes mellitus **
[41]	ANN * DT LR	16,340,661	Numeric	Sensitivity 0.22-0.24 AUC 0.62–0.64	Demographic (age, race, marital status, education, previous terminations, Special Nutritional Program for Women, Infants and Children, pre-pregnancy smoking, body mass index, weight). Obstetric (parity, pre-pregnancy diabetes, gestational diabetes, pre-pregnancy hypertension, gestational hypertension, hypertension eclampsia, previous preterm birth, infertility treatment, infertility medication, Assisted Reproductive Technology, previous cesarean section, Gonorrhea, Syphilis, Chlamydia, Hepatitis C).
[42]	DT LR SVM *	300	Electrohysterogram	Specificity 0.86-1.00 AUC 0.60–0.61	Uterine electrical signals (root mean squares, peak frequency, median frequency, sample entropy)
[46]	LR RNN * SVM	25,689	Text (5,602,792 Medical Concepts)	Sensitivity 0.66-0.97 AUC 0.73–0.83	Twin pregnancy, short cervical length, hypertensive disorder, systemic lupus erythematosus, hydroxychloroquine sulfate
[47]	CNN	157	Magnetic Resonance Imaging	Accuracy 0.94	Increased cerebrospinal fluid and reduced cortical folding due to impaired brain growth

ANN—Artificial Neural Network, AUC—Area under the Receiver Operating Characteristic Curve, CNN—(3-Dimensional) Convolutional Neural Network, DT—Decision Tree, LR—Logistic Regression, NB—Naïve Bayes, RF—Random Forest, RNN—Recurrent Neural Network, SVM—Support Vector Machine, * Method with the Best Performance, ** Predictors Listed Based on the Variable Importance Ranking of the Random Forest.
